# RNA-Seq Transcriptome Profiling of Upland Cotton (*Gossypium hirsutum* L.) Root Tissue under Water-Deficit Stress

**DOI:** 10.1371/journal.pone.0082634

**Published:** 2013-12-06

**Authors:** Megan J. Bowman, Wonkeun Park, Philip J. Bauer, Joshua A. Udall, Justin T. Page, Joshua Raney, Brian E. Scheffler, Don. C. Jones, B. Todd Campbell

**Affiliations:** 1 USDA-ARS, Coastal Plains Soil, Water and Plant Research Center, Florence, South Carolina, United States of America; 2 Clemson University Pee Dee Research and Education Center, Florence, South Carolina, United States of America; 3 Brigham Young University, Provo, Utah, United States of America; 4 USDA-ARS, MSA Genomics Laboratory, Stoneville, Mississippi, USA; 5 Cotton Incorporated, Agricultural and Environmental Research, Cary, North Carolina, United States of America; New Mexico State University, United States of America

## Abstract

An RNA-Seq experiment was performed using field grown well-watered and naturally rain fed cotton plants to identify differentially expressed transcripts under water-deficit stress. Our work constitutes the first application of the newly published diploid D_5_ *Gossypium raimondii* sequence in the study of tetraploid AD_1_ upland cotton RNA-seq transcriptome analysis. A total of 1,530 transcripts were differentially expressed between well-watered and water-deficit stressed root tissues, in patterns that confirm the accuracy of this technique for future studies in cotton genomics. Additionally, putative sequence based genome localization of differentially expressed transcripts detected A_2_ genome specific gene expression under water-deficit stress. These data will facilitate efforts to understand the complex responses governing transcriptomic regulatory mechanisms and to identify candidate genes that may benefit applied plant breeding programs.

## Introduction

 Limited water resources are one of the major environmental pressures impacting global crop production [1]. As climate change and decreases in arable land place increasing strain on available resources, it is essential to develop methods to study abiotic stress and its influence on the growth and development of the world’s major crops. Water-deficit influences a wide range of plant processes, from whole-plant growth and development to the molecular regulation of essential transcriptional pathways, and thus significantly impacts both plant physiology and metabolism. Characteristic responses of water-deficit stress can include wilting, decreased photosynthetic rate [2,3] and stomatal closure [4–6]. These responses negatively impact carbon metabolism. The production of reactive oxygen species (ROS) is also commonly found in water-deficit stressed plant cells, where they may destroy lipids and interact with major cellular signaling pathways [7]. 

 The effects of water-deficit stress to the aerial portions of plants, including leaf, stem and flower tissues, have been well documented [8–10]. Recent research emphasized downstream effects of stresses to the integral root system, responsible for water uptake, on all plant tissues [11]. One root response is altered root architecture that may counter a change in soil properties by decreasing the development of lateral roots [12–14]. Degradation of lateral root amyloplasts is associated with increased hydrotropism in the main root [12,15]. The effect of the plant hormones abscisic acid (ABA), auxin, cytokinins, and gibberellin on root responses during water-deficit stress are also well-documented [8,16–18]. Thus, complex mechanisms contribute to root tissue responses to water-deficit stress [14,19–21]. These mechanisms are mediated by altered gene expression profiles in rice (*Oryza sativa* L.) [22,23], pine (*Pinus pinaster* Ait) [24] and maize (*Zea mays* L.) [25]. 

One crop influenced by the global reduction in available water resources is upland cotton (*Gossypium hirsutum* L.). Cotton is one of the world’s most valuable crops, providing much of the planet’s natural fiber for the global textile industry. Although additional economic value is captured from cottonseed and its associated products, cotton fiber represents about 90% of cotton’s total economic value [26]. China, the United States and India provide most of the world’s cotton, a combined total of more than 15.9 million metric tonnes of cotton lint and 30.4 million metric tonnes of cottonseed, a value of 22.8 billion and six billion dollars in 2011, respectively (FAOSTAT, www.faostat.fao.org). Environmental stresses such as extreme temperatures, soil salinity and water-deficit stress occur in these regions, further exacerbating population pressure as the effects of global climate change continue. Cotton is a warm-climate plant whose aerial tissues have evolved mechanisms conferring moderate tolerance to water-deficit stress [27–29]. An extensive root system also allows the plant to adjust to varying soil moisture levels. Plant breeding for water-deficit tolerance in cotton has resulted in a wide variety of adapted genotypes throughout the world [29–31]. 

 Molecular processes in response to water-deficit stress have been studied at great length in cotton. Studies include the evaluation of global gene expression changes occurring during water-deficit in cultivated tetraploid cotton [18,22,32–34] and the diploid relatives *G. arboreum* L. and *G. herbaceum* L. [2,35–38]. Many of these experiments were conducted using microarray or cDNA-AFLP gene expression approaches. Although a number of significant changes in gene expression resulting from water-deficit stress were identified in these studies, the development of next generation sequencing technologies (NGS) offer opportunities to more accurately quantify those differences [39]. The recent publication of the whole genome sequence of the cotton diploid relative *Gossypium raimondii* Ulbrich [40] has expanded the use of NGS as a tool to study cotton development. 

In this study, we report the first application of the diploid *G. raimondii* whole genome sequence and Illumina NGS technology to pursue RNA-seq analysis of global gene expression changes in field grown tetraploid cotton root tissue. Several genes and major biochemical pathways were up regulated in root tissue under water-deficit stress, confirming the success of this technique for transcriptome evaluation of tetraploid cotton species. Using NGS to assess global gene expression patterns in polyploid plant species is complicated; short reads found in several related loci can align to a single transcript or be removed from analysis, impacting accurate quantification of expression levels [41]. Gene duplication and genome reorganization events contribute to such complexity. In order to minimize the effects of genome complexity, we used the new PolyCat annotation pipeline [42] which assigns putative genome localization for many of the identified differentially expressed transcripts. Our objective was to use NGS to measure global gene expression profiles in field-grown tetraploid cotton root tissues under water-deficit stress to identify candidate genes for future research in molecular cotton breeding. Our results will provide an improved understanding of the putative transcriptional mechanisms involved in root responses to water-deficit stress in this important global crop. 

## Materials and Methods

### Ethics Statement

The field studies did not involve any human, animal, or endangered species. The corresponding author is an Adjunct Professor with North Carolina State University and has unrestricted access to field research facilities.

### Plant Materials

Root tissues from *G. hirsutum* cultivar ‘Siokra L-23’, selected for its previously established high level of water-deficit tolerance [27–29], were collected from field-grown plants under water-deficit and well-watered conditions at the North Carolina State University Sandhills Research Station near Jackson Springs, NC, USA according to the method described by 18. Roots were collected from three independent plants within each of the two water treatments. Samples were harvested during the third week of flowering on a single sample date, when significant differences in xylem water potential of the uppermost fully expanded leaves between treatments occurred, as determined by a pressure bomb (Model 600, PMS Instrument Company, Albany, OR). Plants were considered water-deficit stressed when leaf water potentials were -2.0 MPa or greater and well-watered when leaf water potentials were -1.9 MPa or lower [43]. Average water potential of well-watered and water-deficit stressed plants is presented in Table 1. Total RNA was isolated as previously described [18,44] using the XT buffer system with the addition of chloroform/iso-amyl alcohol extraction and LiCl precipitation steps [45]. 

**Table 1:** Leaf water potential values of upland cotton plants used in RNA-seq evaluation.

**Treatment**	**Plant**	**Leaf water potential (MPa**)
Well-watered	1	-1.60
	2	-1.35
	3	-1.45
Water-deficit	1	-2.20
	2	-2.70
	3	-2.85

Leaf water potential values of selected upland cotton plants from both well-watered and water-deficit treatments.

### RNA-Seq library construction and sequencing

Six individual barcoded libraries were created with the Illumina RNA TruSeq kit (Illumina) as per manufacturer's instructions using 2 µg of total RNA from three individual root RNA samples for each treatment. Library quality was assessed with an Agilent Bioanalyzer 2100 (Agilent) and the concentration of each individual library was calculated using qPCR. Libraries were pooled together so that each barcode was represented in equimolar amounts and sequenced in a single lane of 50bp Illumina HiSeq 2000. 

### Read trimming and mapping

Reads were trimmed with Sickle (https://github.com/najoshi/sickle) with a quality cutoff of 20. Genomic Short-Read Nucleotide Alignment Program (GSNAP) [46] was used to map reads to the *G. raimondii* 2.1 whole genome reference sequence [40], with SNP-tolerant mapping using a SNP index based on deep coverage of *G. arboreum* and *G. raimondii*, as described in [42]. The "-N 1" option was used for GSNAP to identify novel splice sites. Putative A_T_ and D_T_ genome localization of the differentially expressed transcripts was conducted using the PolyCat pipeline [42] which categorizes and maps DNA sequence reads of allotetraploid *G. hirsutum* to progenitor diploid genomes of *G. arboreum* (A_2_) and *G. raimondii* (D_5_) [47–49]. 

### Differential expression

The total count of mapped reads for each library was converted to a CSV file and imputed into the DESeq (Version 1.9.12) package in R to test for significant differential expression between water-deficit and well-watered treatments [50] using a false discovery rate (FDR) of 5%. Data quality analysis was conducted by calculating and visualizing the Euclidean distance and principle component analysis of well-watered and water-deficit treatment samples using the DESeq (Version 1.9.12) package. Read count data was deposited in the National Center for Biotechnology Sequence Read Archive (NCBI SRA) (Accession No. PRJNA210770) and will be made available through CottonGen. 

### Functional annotation

Significant differentially expressed transcripts identified by DESeq analysis, and additional splice variants identified from the *Gossypium raimondii* v. 2.1 sequence [40] in Phytozome [51], were further evaluated for functional gene ontology annotation using default parameters in Blast2Go software [52]. Annotation was enhanced by merging the output of an additional InterProScan [53] analysis with the initial BLAST annotation so that additional transcripts without initial gene ontology association could be functionally characterized. The Blast2GO ANNEX program and an optional validation step were used to confirmed sequence annotation for each transcript [52]. Gene ontology enrichment analysis was conducted using AgriGO [54]. Differentially expressed transcripts with Kyoto Encyclopedia of Genes and Genomes (KEGG) Orthology IDs provided by Phytozome were mapped to specific pathways using the “Search and Color” Pathway tool, searching against the reference pathway (KO). 

### RT-qPCR RNA extraction and cDNA synthesis

Due to limited tissue amounts of the samples used for RNA-seq, root tissues were harvested from additional plants grown in the same plots and experimental conditions. Tissues were flash frozen in liquid nitrogen and stored at -80°C until being processed for RNA extraction. Individual root tissues from each plant per treatment were homogenized in liquid nitrogen and total RNA was extracted from 100 mg of homogenized tissue using the Spectrum Plant Total RNA kit with the On-Column DNase I Digest Set column DNAse (Sigma Aldrich) according to the manufacturer’s protocol. RNA was quantified using a NanoDrop spectrophotometer (ThermoFisher Scientific) and quality was examined using the Bioanalyzer 2100 (Agilent, Santa Clara, CA). A no-reverse transcription control on all RNA samples was used to determine DNA contamination using *G. hirsutum* alpha-tubulin (*TUA11*) gene (Gorai.010G125700), a reference gene identified from the RNA-seq data in this study. cDNAs were synthesized from 1 µg of total root RNA using the SuperScript® III First-Strand Synthesis SuperMix (Invitrogen) kit according to the manufacturer’s specifications. cDNA was diluted 10-fold for use in RT-qPCR reactions. Synthesized cDNAs were stored at -20°C.

### RT-qPCR

Transcript sequences from *G. raimondii* were used in NCBI BLAST to identify the closest *G. hirsutum* sequence for primer design. Primers were designed using NCBI-Primer BLAST and diluted to a concentration of 5µM (Table 2). Template DNA for primer efficiencies was obtained through PCR using the primers specifically designed for RT-qPCR. PCR products were purified from agarose using the Wizard SV Gel and PCR Clean Up System (Promega) and DNA was quantified using the Qubit® dsDNA HS Assay Kit (Invitrogen). Two independent 10-fold dilutions of DNA for each dilution series were split into three reactions (12.5 µl per well). RT-qPCR was performed using Maxima SYBR Green/Rox qPCR Master Mix (2X) and the iCycler PCR Detection System with the standard two-step amp + melt protocol (Bio-Rad). Efficiencies were calculated using the protocols as described by 55,56. 

**Table 2:** RT-qPCR target and reference gene primers for RNA-seq confirmation.

**Genename**	***G. raimondii* transcript name**	***G. hirsutum* sequence with greatest homology**	**Forward (5'-3')**	**Reverse (5'-3')**
GhPIP1;8	Gorai.013G019300	*Gossypium hirsutum* cultivar des119 aquaporin PIP1;8	GTTTTCAGAGAGGCAACCTA	CCCAGCTCTATAAAAGGACC
GhAQP1	Gorai.003G158100	*Gossypium hirsutum* aquaporin 1 (AQP1)	TGGTTGTTAAGTGGGTGAAA	TAGTCCTTGTCTGTTTGAGC
GhNIP6;1	Gorai.009G124500	*Gossypium hirsutum* aquaporin (NIP6.1)	TCTCACTCACAAGAAAGGTG	ATCAGAGTTTCAGAGCCTTG
GhPIP2;8	Gorai.009G107200	*Gossypium hirsutum* PIP protein (PIP2;8)	ATTTGTGGTTGTGGGTTAGT	CAACCCAGTTCCCTTATTGA
GhTIP2;3	Gorai.003G064000	*Gossypium hirsutum* cultivar TM-1 aquaporin TIP2;3	GCATCTTTTACTGGATTGCC	GATGATCTCCATCACCACTC
GhPOD6	Gorai.012G141300	*Gossypium hirsutum* bacterial-induced class III peroxidase (pod6)	GCTCGTGATTCTGTAGTTCT	CTGCAAATTTTTGCTTCTGC
GhPOD9	Gorai.004G265900	*Gossypium hirsutum* POD9 precursor (pod9)	CAAACACACTCAAACAACGA	TCTTGGTCTGTTTGAAGCAA
GhLea3	Gorai.007G199900	*Gossypium hirsutum* dehydrin (Lea3-D147) gene	GGACTGAAACAGAGGCTAAA	CCATCACTCCTTTCTTCTCG
GhCloMX	Gorai.002G078800	*Gossypium hirsutum* clone MX019A11-jhj	ATCAGGCTTAGAAACACAGG	ATCTTCCTTTCCATGTTCCC
GhLOX1	Gorai.006G238200	*Gossypium hirsutum* bacterial-induced lipoxygenase (Lox1)	ATCCTATCAAGGCATTCGTC	TCTCTACAATCCGTTCCTCT
GhTHIA	Gorai.009G176400	*Gossypium hirsutum* thiazole biosynthetic enzyme	ATGGACATGATCACCTATGC	AACAGACTGCTCGACAATAG
GhTUA11	Gorai.010G125700	*Gossypium hirsutum* alpha-tubulin (TUA11)	TTGGGATCTTTGTTGTTGGA	GTTCAAGAAGCGAATGAGTG

Target and reference gene primers for RT-qPCR confirmation of RNA-seq results in tetraploid upland cotton. Primers were designed using NCBI-BLAST and diluted to 5 μM with annealing temperatures at 55 °C.

To confirm that each primer set used in RT-qPCR was accurately amplifying the correct *G. hirsutum* sequence, each purified PCR amplicon was cloned using either the TOPO Zero Blunt or TA Cloning Systems (Invitrogen), with OneShot Top10 competent cells (Invitrogen). PCR amplification using T3/T7 primers was used to confirm fragment insertion and correct orientation. Four individual colonies were bi-directionally sequenced as previously described in [44]. Sequence evaluation of inserted amplicons was conducted with Geneious software version 6.1 (Biomatters Ltd.) and homology-based BLAST search of amplicons was used to confirm sequence identity. 

 RT-qPCR was performed in duplicate 12.5 µl volumes with cDNAs from two independent cDNA superscript reactions and Maxima SYBR Green qPCR Master Mix (Fermentas). All reactions were analyzed with the iCycler Real Time PCR Detection System (Bio-Rad) with the default two-step amplification plus melt curve protocol for each reaction (Bio-Rad). Target transcripts included mRNA from ten genes identified as differentially expressed between water-deficit and well-watered treatments according the DESeq analysis of RNA-seq data. Transcript Gorai.012G141300 was selected internally from the RNA-seq data as a reference transcript and validated using the RefFinder program http://www.leonxie.com/referencegene.php). Relative Expression Ratios (RER) were calculated using the ΔCt method [55]. RT-qPCR protocols followed the MIQE guidelines [57]. 

## Results

### Total number of sequenced reads

**Figure 1:**
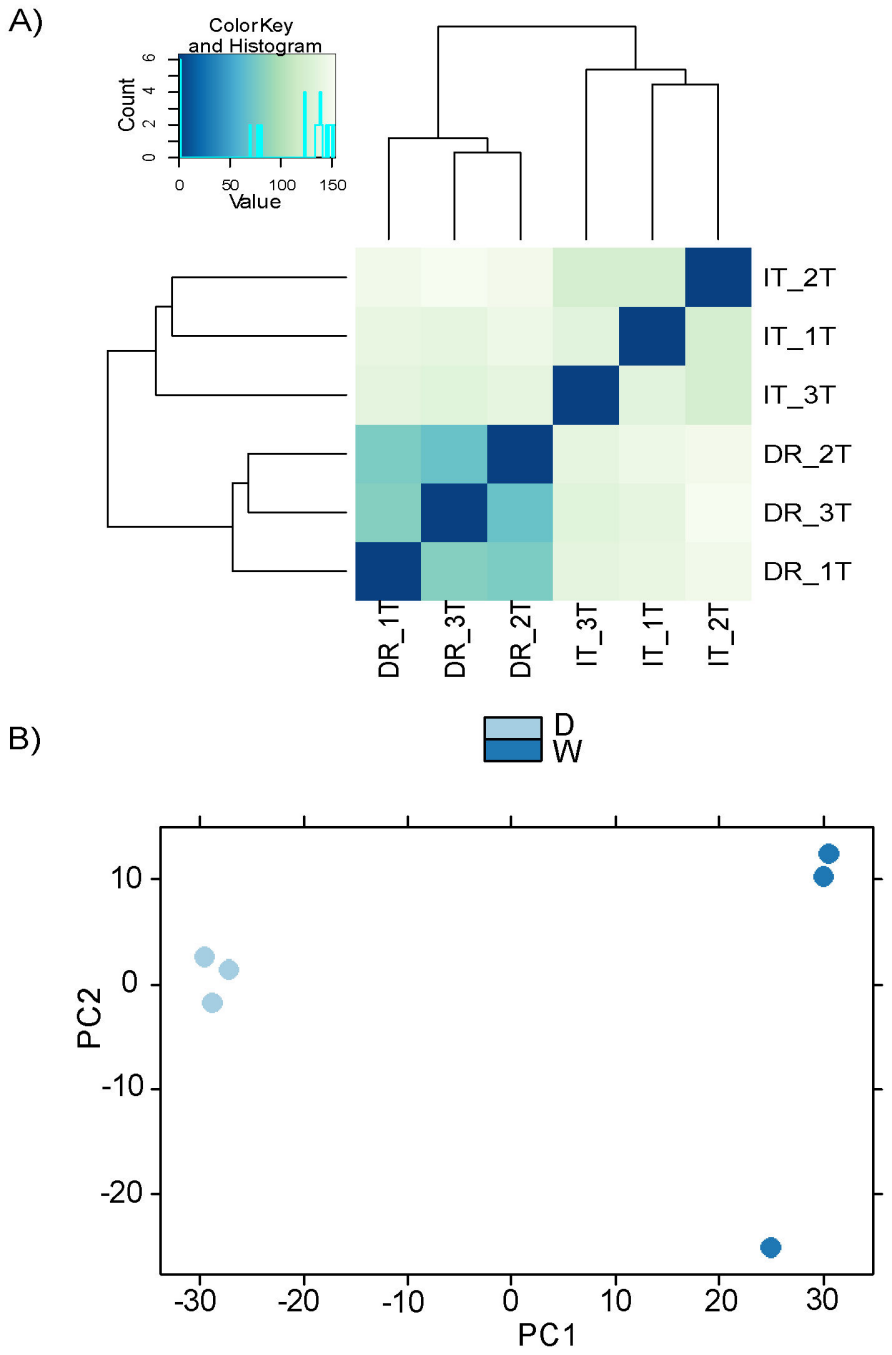
Data quality evaluation of mRNA-seq data. A) Measurement of Euclidean distances and B) Principle Component Analysis (PCA) of all samples to assess data quality. Color key indicates level of similarity between libraries. Analysis was conducted using DESeq (Version 1.9.12) [50]. .

To assess global transcriptome changes occurring in the root tissue of tetraploid upland cotton during water-deficit stress, total RNA samples from three individual cotton plants of the cultivar Siokra L-23 under both water-deficit and well-watered conditions were used to create six independent libraries that were sequenced using the Illumina HiSeq 2000 sequencing platform. Approximately 109.6 million 50 bp reads from all six libraries were trimmed with Sickle and mapped to 33,930 transcripts from the *G. raimondii* 2.1 whole genome reference sequence [40]. Over 90% of identified transcripts had between 0 and 1000 mapped reads. Fifty percent of transcripts had fewer than 100 mapped reads, 50% of transcripts had more than 100 reads, and 7 % had more than 1000 mapped reads. Data quality was assessed using heatmap visualization of Euclidean distances and principle component analysis of all samples, conducted using DESeq (Version 1.9.12) [50] (Figure 1).

### Global Transcriptome Changes during Water-Deficit Stress

**Figure 2:**
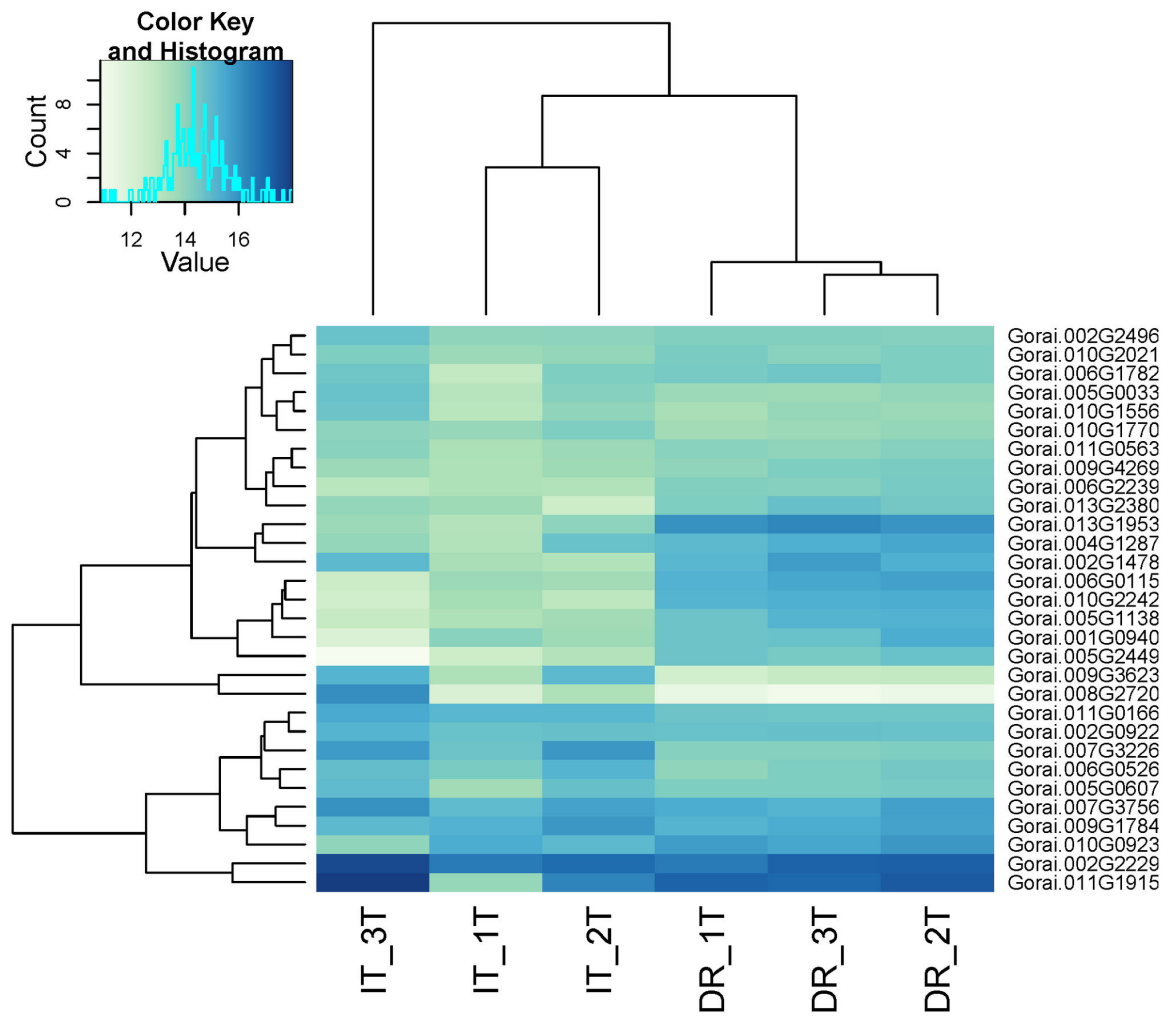
Visualization of thirty most differentially expressed genes. Hierarchical clustering and heatmap visualization of the thirty most differentially expressed genes between well-watered and water-deficit treated upland cotton root samples, using variance stabilization with a FDR of %5 using DESeq (Version 1.9.12) [50]. Color key indicates transcript abundance for each gene.

**Figure 3:**
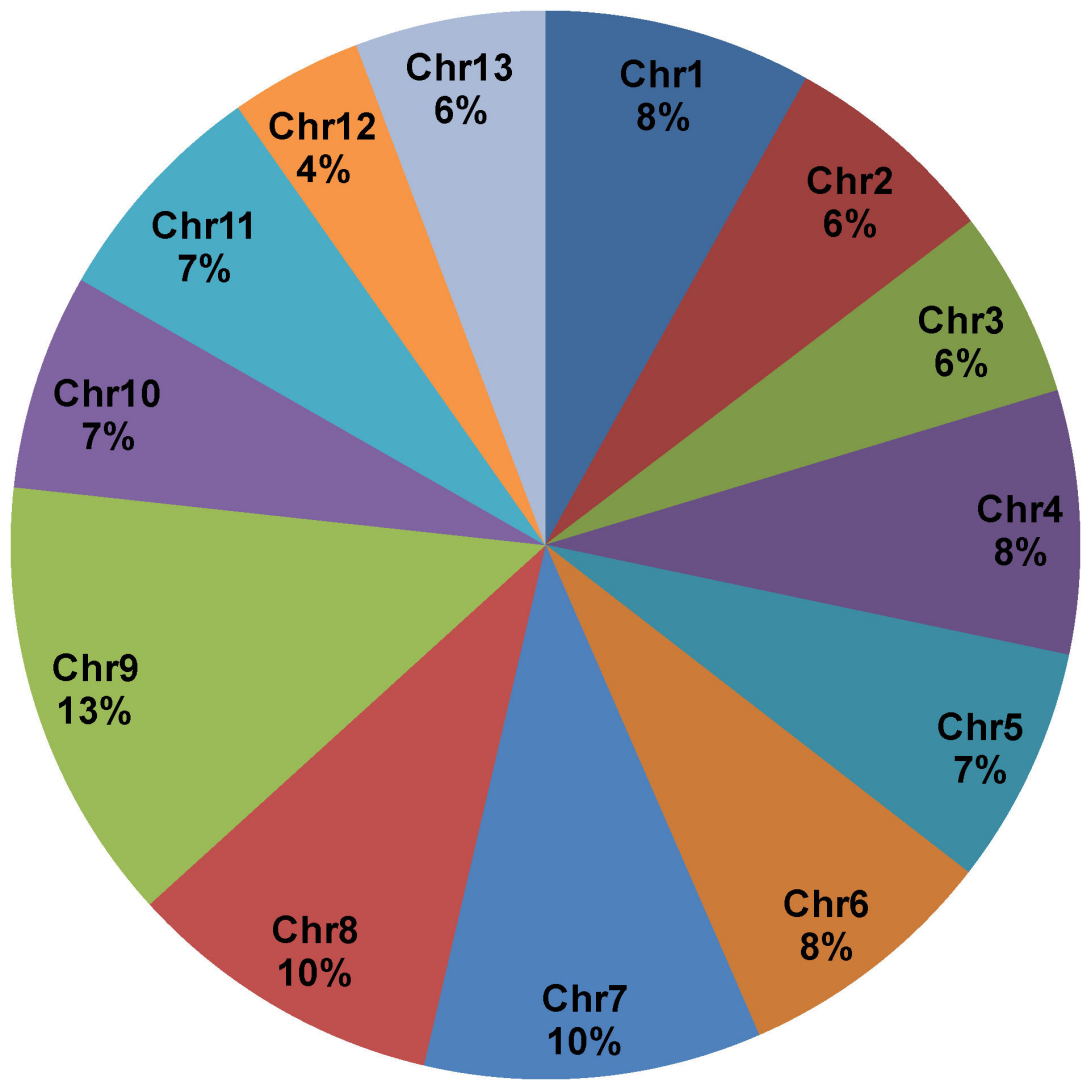
Chromosomal distribution of all differentially expressed transcripts. Distribution of differentially expressed transcripts, as determined by the alignment of *G. hirsutum* RNA-seq reads to the thirteen chromosomes of diploid relative *G. raimondii*.

The total number of mapped sequenced reads for all identified transcripts was used for differential expression analysis in DESeq with an FDR of 0.05. A total of 1530 genes were either up or down regulated between water-deficit and well-watered upland cotton root samples. Of those 1530 genes, 913 were up-regulated under water-deficit stress and 617 down-regulated. A subset of differentially expressed genes is shown in Figure 2. Differentially expressed genes were distributed across all 13 chromosomes in the diploid progenitor genome of *G. raimondii*, determined by Gorai transcript IDs provided by Phytozome [40] (Figure 3). Several genes identified by a previously published gene expression study using cDNA-AFLP [18] were also found by RNA-seq. Although the total number of differentially expressed genes was different between the studies (304 in cDNA-AFLP and 1530 in RNA-seq), similar transcripts were involved in water uptake, heat stress and carbohydrate metabolism, including aquaporin water uptake protein PIP 1;3, Heat Shock Protein 26, and mannose-6-phosphate isomerase. 

### Functional annotation of differentially expressed transcripts

Following differential expression analysis with DESeq, all significant transcripts and associated splice variants, a total of 2942 transcripts, were annotated using the Blast2Go application [52] . Of the 2942 total sequences, 2416 were successfully annotated; 102 genes were not analyzed because they exceeded the maximum size allowance (>8000bp) and 74 had no sequence homology in BLAST. After enhancing the annotation by including the results of an InterProScan database search and the ANNEX augmentation procedure, 112 additional annotations were added and 1821 annotations were confirmed. 

### Genome localization of differentially expressed transcripts

NGS and gene expression analyses are complicated in polyploid plants [58–61]. In order to putatively identify genome localization of the 1530 differentially expressed transcripts identified by RNA-seq, we used the PolyCat read mapping pipeline [42]. PolyCat uses SNPs identified between the related diploid genomes of *G. arboreum* (A_2_) and *G. raimondii* (D_5_) to map total NGS reads to either the A genome (A_T_) or D genome (D_T_) of the allopolyploid (AD_1_) *G. hirsutum*. Genes up-regulated under water-deficit predominately contained A_T_ genome specific reads, with 407 (44.6%) of both the water-deficit and comparative well-watered transcripts having a majority of reads that mapped to the A_T_ genome. Genes down-regulated in water-deficit were more evenly distributed, where transcripts from both treatments were comprised of reads mapping to either the A_T_ (225, 36.5%) or D_T_ (217, 35.4%) genomes. Only two (0.2%) of the up-regulated transcripts contained reads mapping only to the A_T_ genome, and three (0.3%) transcripts contained reads mapping only to the D_T_ genome. Only five (0.8%) genes down-regulated under water-deficit stress had reads that mapped to the A_T_ genome only, and five transcripts were comprised of reads that mapped only to the D_T_ genome. Of the total number of differentially expressed transcripts identified by RNA-seq, 101 could not be associated with a specific genome within tetraploid cotton (Table 3). 

**Table 3:** Putative genome localization of transcripts according to the PolyCat annotation pipeline.

**Total up-regulated transcripts**	**A_T_ both**	**D_T_ both**	**A_T_ Water-Deficit, D_T_ Well-Watered**	**D_T_ Water-Deficit, A_T_ Well-Watered**	**A_T_ only**	**D_T_ only**	**None**
913	407	315	72	74	2	3	40
Percentage of Total	44.6	34.5	7.9	8.1	0.2	0.3	4.4
**Total down-regulated genes**	**A_T_ both**	**D_T_ both**	**A_T_ Water-Deficit, D_T_ Well-Watered**	**D_T_ Water-Deficit, A_T_ Well-Watered**	**A_T_ only**	**D_T_ only**	**None**
617	225	217	50	54	5	5	61
Percentage of Total	36.5	35.2	8.1	8.8	0.8	0.8	9.9

Putative genome localization of water-deficit stressed and well-watered associated transcripts based on SNP evaluation and comparison to diploid progenitor genomes, according to the PolyCat annotation pipeline [42]. “Both” denotes transcripts in which both water-deficit stressed and well-watered reads predominantly mapped to a specific genome, “A_T_ Water-Deficit, D_T_ Well-Watered” and “D_T_ Water-Deficit, A_T_ Well-Watered” denote those transcripts in which the predominant genome differed by treatment, “A_T_ only and D_T_ only” denote genes for which all reads mapped to a specific genome for both treatments. “None” denotes transcripts that could not be associated with a specific genome.

### Gene ontology

Gene ontologies most highly represented for molecular function were “catalytic activity (1176)”, “binding (1196)”, “transporter activity (164)” and “nucleic acid binding transcription factor activity (189)”. Gene ontologies for cellular component were “cell (1664)”, “organelle (1142)” and “membrane (720)”. Gene ontologies for biological process were “metabolic process (1317)”, “cellular process” (1371), and “response to stimulus” (871). Not surprisingly, many of the enriched biological process ontology terms were “response to temperature stimulus (*P*-value: 1.40E-10), “response to high light intensity” (*P*-value: 1.70E-09), “response to heat” (*P*-value: 4.40E-09) “response to water (*P*-value: 3.2E-05)” and “response to water depravation (*P*-value: 6.2E-05)”. Enriched molecular function terms were “glycogen debranching enzyme activity” (*P*-value: 7.80E-08), “hydrolase activity, hydrolyzing O-glycosyl compounds” (*P*-value: 0.00022), “transcription regulator activity” (*P*-value: 0.00035) and “hydrolase activity, acting on glycosyl bonds” (*P*-value: 0.00064). Enriched cellular component were “protein serine/threonine phosphatase complex” (*P*-value: 0.0048), “stromule” (*P*-value: 0.0066) and “anchored to membrane” (*P*-value: 0.015).

### KEGG pathway analysis

**Figure 4:**
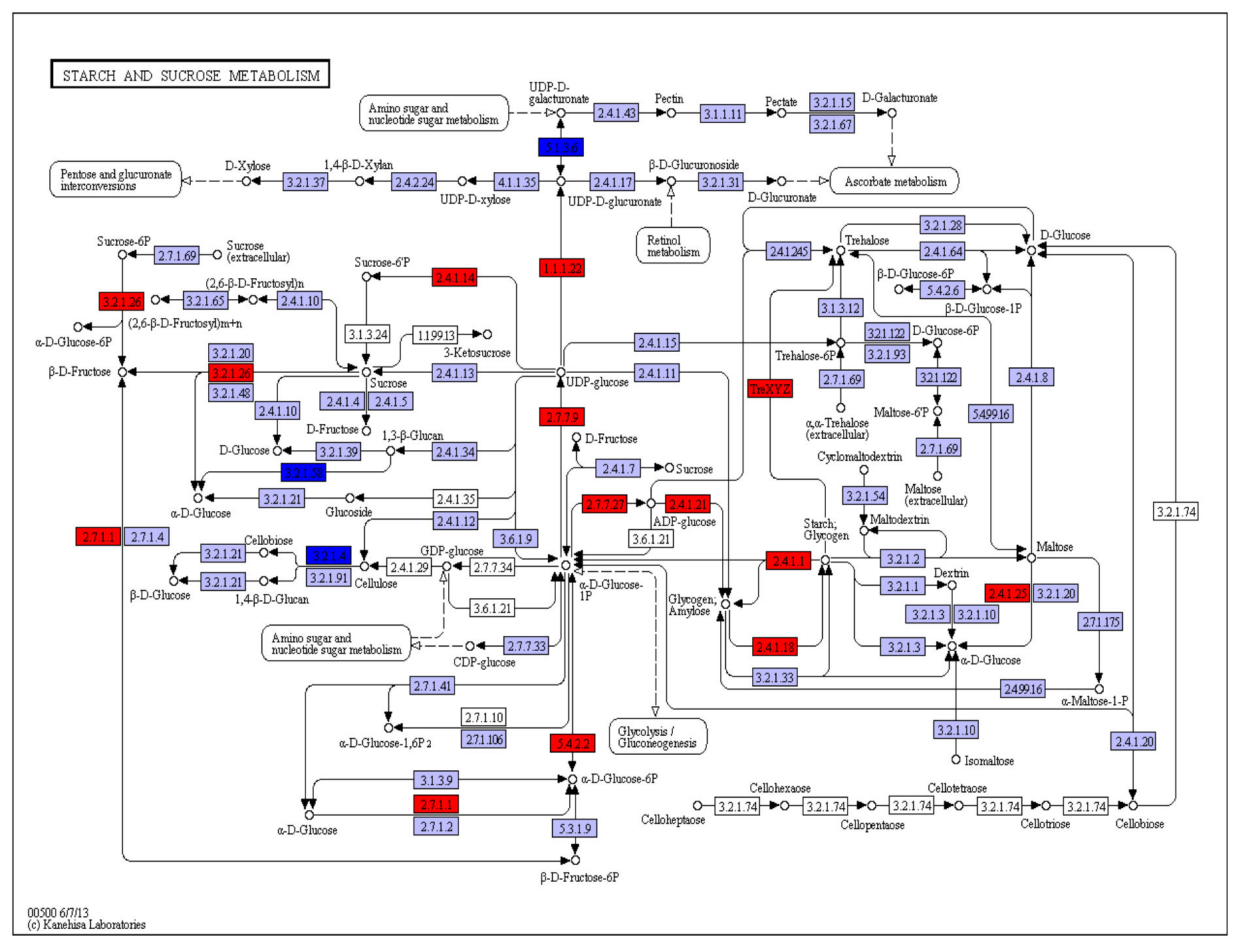
KEGG pathway visualization of starch and sucrose associated differentially expressed transcripts. KEGG search and color pathway analysis of significant differentially expressed transcripts in starch and sucrose metabolism pathways in upland cotton root under water-deficit stress. Enzymes coded red are up regulated under water-deficit, blue genes are down regulated, and purple genes denote the reference pathway.

The primary pathways impacted in root tissue of upland cotton plants during water-deficit stress were starch and sucrose metabolism (109 sequences, 23 enzymes), glycolysis-gluconeogenesis (37 sequences, 11 enzymes), amino sugar and nucleotide sugar metabolism (35 sequences, 14 enzymes), and galactose metabolism (31 sequences, 14 enzymes). Other major plant pathways impacted included flavonoid biosynthesis (15 sequences, 6 enzymes), carotenoid biosynthesis (9 sequences, 2 enzymes), and oxidative phosphorylation (8 sequences, 2 enzymes) (Figure 4). 

### RT-qPCR of specific genes of interest

**Figure 5:**
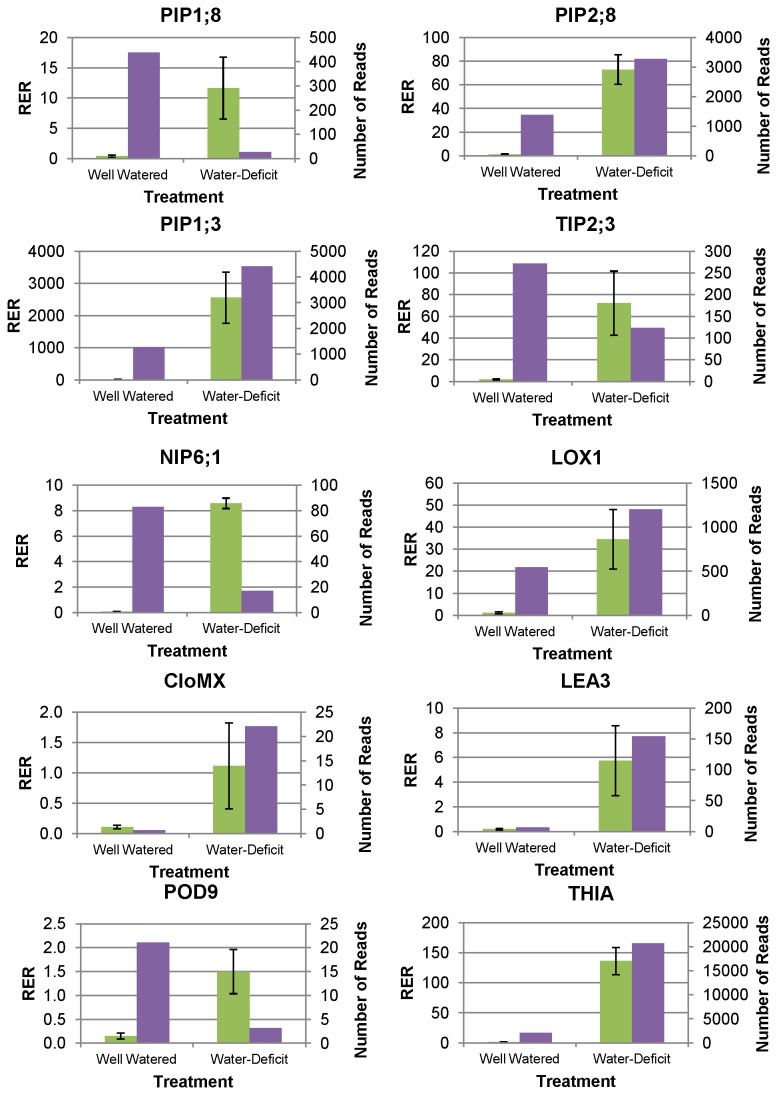
RT-qPCR confirmation of RNA-seq results. RT-qPCR confirmation (left Y-axis, green bars) and normalized RNA-seq read count values (right Y-axis, purple bars) of differentially expressed genes between well-watered and water-deficit treatments. RT-qPCR was calculated using the **Δ**Ct method [55]. Error bars represent standard error of genotype means.

To further investigate the expression patterns of specific genes of interest from the RNA-seq experiment, we conducted RT-qPCR using 10 transcripts, representing a range in the number of reads per transcript, that were associated with specific aspects of stress physiology and water transport. Transcripts were also selected based on availability of *G. hirsutum* EST sequence, to simplify primer design and amplification in tetraploid cotton. Of the 10 transcripts selected, six (60%) were expressed in the pattern identified by RNA-seq. These six encoded thiazole biosynthetic enzyme (*THIA*), plasma membrane intrinsic aquaporin proteins PIP1;3 and *PIP*2.8, dehydrin (*LEA*3-D147), clone MX019A11-jhj (*CLOMX*), and bacterial-induced lipoxygenase (*LOX1*). In contrast, four genes (40%) had RT-qPCR gene expression patterns that differed from RNA-seq results, and were not differentially expressed between treatments. These were aquaporins PIP1;8, *NIP*6;1, and *TIP*2;3 and peroxidase precursor *POD*9. Overall, a majority of transcripts selected for RT-qPCR were expressed in the same manner as identified using RNA-seq (Figure 5). 

## Discussion

Cotton is a major crop with global economic significance that requires specific environmental factors suitable for plant growth, development and production. As global climate change continues to increase demand for the world’s water resources, it is essential to identify approaches to improve our understanding of how crop plants including cotton respond to water-deficit stress. In the present study, we applied NGS technology to profile transcriptome changes in root tissue of upland cotton undergoing field water-deficit stress. A total number of 1530 differentially expressed genes were identified. In contrast from a previous study that found more genes to be down-regulated [18], most transcripts were up-regulated under water-deficit stress. To our knowledge, this is the first published use of the *G. raimondii* whole genome sequence and RNA-seq to measure transcriptome differences in field grown, tetraploid cotton. With this approach we have identified gene expression changes in root tissues under water-deficit stress, and many will serve as potential targets for future research and the development of molecular breeding tools for cotton breeding programs. 

### Many biochemical pathways are associated with root response to water-deficit

Results of the present study generally confirm previously identified biochemical mechanisms modulating the adaptation of cotton to water-deficit stress and demonstrate the utility of our methods in cotton genomics. Specifically, the induction we observed in genes associated with starch and sugar metabolism is similar to results obtained by other researchers [18,22,32–34] (Figure 4). As root tissues undergo water-deficit stress, increases in carbohydrate metabolism and other osmolyte concentrations alter the osmotic potential of the cell [9]. Water-deficit also induces several hormone responses, including cytokinins, auxin and abscisic acid [8,16–18]. Abscisic acid is an important signaling molecule in the development of root system architecture under water-deficit [12,14,62]. Genes upstream and within the abscisic acid pathway can be up-regulated under water-deficit and one gene specifically, β-carotene hydroxylase, has been shown to be induced under water-deficit stress [63]. The detection of the up regulation of the carotenoid biosynthesis ABA precursor gene 9-*cis*-epoxycarotenoid dioxygenase (NCED) and ABA pathway gene ABA 8'-hydroxylase in this RNA-seq data set identifies target candidate genes for further studies of water-deficit tolerance in the root system of upland cotton. 

 Other plant responses to water-deficit stress involve proteins responsible for cellular water uptake. Aquaporins are a large major intrinsic protein family consisting of 71 members in cotton [44] that have been shown to facilitate the movement of water and other small molecules across cell membranes [18,44,64]. Our results confirm differential aquaporin gene expression in response to water-deficit stress, as has been observed in many plant species, including cotton [18,65–68]. Specifically, we observed differential expression of aquaporin genes in both RNA-seq and RT-qPCR. These examples serve as additional evidence for the potential role of aquaporin expression in mediating water deficit stress tolerance in cotton root tissues. 

### Putative genome localization of water-deficit related genes in tetraploid cotton

 Many agriculturally important plant species, such as wheat (*Triticum aestivum* L.), potato (*Solanum tuberosum* L.), and sugarcane (*Saccharum officinarum* L.) are polyploid [60]. Polyploidzation makes NGS technologies (such as RNA-seq) challenging. NGS depends on read mapping in which large amounts of nucleotide sequence are associated with genome localization; however, due to gene duplication and genomic restructuring events common in polyploids, it is difficult to accurately map reads to their genome of origin [41,42,58,69]. The development of annotation pipelines capable of assigning tetraploid transcript reads accurately to related diploid genomes is a significant improvement in the effort to assess gene expression in polyploidy plant species. In this study, we employed the use of PolyCat, a new NGS annotation pipeline capable of assigning reads from tetraploid *G. hirsutum* (AD_1_) to progenitor diploid A_2_ genome *G. arboreum* and D_5_ genome *G. raimondii*. Putative genome localization was provided by the comparison of SNP data from the sequence of the progenitor genomes to the NGS reads created by this study. 

Gene expression responses to water-deficit stress have been studied extensively in a variety of cotton tissues [18,28,29,32,35,36]. While informative, to our knowledge, no previous study has reported potential genome specific responses to water-deficit. A majority of transcript reads mapped to the A_T_ genome among genes that were up-regulated in response to water deficit; while genes that were down-regulated were represented evenly by both the A and D genomes. The up-regulation of A_T_ genome-specific transcripts indicates the importance of the A genome diploid relative *G. arboreum* in water-deficit response, which has been previously identified to be a source of other stress-related genes [36–38]. Interestingly, transcripts from several genes that were either up- or down-regulated under water-deficit stress had reads that mapped to a single genome. Further investigation of these genome-specific transcripts is called for among A_T_ and D_T_ specific responses to water-deficit stress in tetraploid cotton.

### Future considerations for transcriptome evaluation in tetraploid cotton

This study represents the first reported use of NGS technologies in combination with the recently published *G. raimondii* sequence to evaluate differential transcriptome profiles in upland cotton. Over 1500 genes were differentially expressed between water-deficit stress and well-watered root treatments. Expression patterns for genes associated with sugar metabolism, ABA synthesis, and water uptake were similar to those found in other published reports of gene expression analyses under water-deficit stress. Substantial up- regulation of genes associated with water-deficit, including those associated with responses to changes in temperature, high light intensity, heat, and water was detected in concert with gene ontology enrichment analysis with AgriGO [54]. Transcriptome profiling of tetraploid cotton using the *G. raimondii* published sequence successfully detected global gene expression changes during water-deficit stress. However, further considerations should be made when choosing genes for RT-qPCR analysis, as a majority of genes assayed by RT-qPCR had very low read count values. Very low read counts may exceed the level of accurate detection by the RT-qPCR or by other statistical methods [70–72]. Additionally, evaluating highly- conserved gene families, such as the aquaporins, may be more complicated due to gene duplication and sequence similarity, and this should be considered prior to evaluation [41]. Further comparison of RNA-seq studies using alignments to both the *G. raimondii* diploid genome and *G. hirsutum* transcriptome sequence, as it becomes more available, will be of significant interest. 

## Conclusions

Differentially expressed transcripts were associated with the up-regulation of important biochemical pathways needed for cellular osmotic balance, abscisic acid and cellular water uptake. Similar results from water-deficit stress research with microarray and cDNA-AFLP confirm the use and accuracy of this technique for future research in cotton genomics. An additional analysis of genome localization based on available SNP data identified A_T_ up regulation of genes in response to water-deficit, the first discussion of a high throughput sequencing approach to quantify water-deficit responsive genome expression patterns within tetraploid cotton. Results from this study advance our current understanding of water-deficit response in the root tissue of upland cotton, opening new areas of research in cotton breeding and genomics.
